# Short term elevation in dietary protein intake does not worsen insulin resistance or lipids in older adults with metabolic syndrome: a randomized-controlled trial

**DOI:** 10.1186/s40795-017-0152-4

**Published:** 2017-04-17

**Authors:** Il-Young Kim, Scott E. Schutzler, Gohar Azhar, Robert R. Wolfe, Arny A. Ferrando, Robert H. Coker

**Affiliations:** 1 Department of Geriatrics, Center for Translational Research in Aging & Longevity, Donald W. Reynolds Institute on Aging, University of Arkansas for Medical Sciences, Little Rock, AR USA; 2 Institute of Arctic Biology, University of Alaska Fairbanks, 902 North Koyukuk Drive, Fairbanks, AK 99775-7000 USA

**Keywords:** Stable isotope tracers, Liver, Muscle, Insulin resistance, Protein intake

## Abstract

**Background:**

There is a great deal of controversy as to whether higher protein intake improves or worsens insulin sensitivity in humans. The purpose of the study was to determine the influence of a short-term elevation in dietary protein on hepatic and peripheral insulin sensitivity in twelve older subjects (51–70 yrs) with metabolic syndrome.

**Methods:**

Individuals were randomly assigned to one of the dietary groups: recommended protein intake (RPI, 10% of daily calorie intake) or elevated protein intake (EPI, 20% of daily calorie intake) for 4 weeks. Prior to and immediately following the dietary intervention, subjects were studied with primed continuous infusion of [6,6-^2^H_2_]glucose and [1-^13^C]glucose dissolved in drink during the dual tracer oral glucose tolerance test (DT OGTT) to determine hepatic and peripheral insulin sensitivity. Plasma lipids were measured pre- and post-dietary intervention.

**Results:**

In both intervention groups: 1) hepatic insulin sensitivity as assessed by the endogenous glucose rate of appearance (glucose R_a_), 2) peripheral insulin sensitivity as assessed by the metabolic clearance rate of glucose normalized to plasma glucose concentration (MCR) and/or the rate of glucose utilization (R_d_) or 3) glucose/insulin AUC were unaffected by the diets. Moreover, fasting lipid was not affected by RPI or EPI.

**Conclusion:**

Our findings suggest that a short-term elevation in EPI with correspondingly higher branched chain amino acid (BCAA) contents has no detrimental impact on hepatic and peripheral insulin sensitivity or plasma lipid parameters in older adults with metabolic syndrome.

**Trial registration:**

ClinicalTrials.gov Identifier: NCT02885935; This trial was registered retrospectively (Study start date, April 01, 2013, date of registration, August 26, 2016).

**Electronic supplementary material:**

The online version of this article (doi:10.1186/s40795-017-0152-4) contains supplementary material, which is available to authorized users.

## Background

It has been well demonstrated that dietary protein intake above the recommended dietary allowance (RDA) of 0.8 g protein/kg body weight/day which promotes reductions in body fat mass due in part to increased satiety and/or energy expenditure linked to feeding induced thermogenesis [1]. A high protein diet has also been linked to improvements in lean body mass (reflecting muscle mass) via the stimulation of net protein synthesis [2, 3], which has also been accompanied by improved strength and function [4]. Given that skeletal muscle is the largest organ and responsible for the majority of postprandial glucose disposal [5], an increase in protein intake may promote the preservation of skeletal muscle and lead to improvements in insulin sensitivity [6]. Studies have also shown that increased protein intake may have favorable effects on circulating triglyceride concentrations [7]. Indeed, elevations in protein intake have been linked to many improvements in what many consider the hallmarks of metabolic syndrome (i.e., hypertension, atherosclerosis, hyperlipidemia) [8]. Considering these data, one would anticipate benefits in metabolic health by increasing the amount of protein and decreasing the amount of calories from fat (particularly, saturated fats) and highly processed carbohydrates.

Despite the potentially favorable influence of higher protein intake on metabolic health, controversy exists regarding the results (improved or worsen) from short- (<6 months) and long-term intervention studies and/or the phenotype of participants that includes those who are healthy, obese, insulin resistant, and/or have type 2 diabetes [9]. Moreover, the term itself “high protein” can be used to indicate a slight elevation in protein intake or a diet comprised of only dietary fat and protein [10]. Despite these wide variations in protein intake that obviously affect dietary intake of fat and carbohydrate, studies based on dietary intake data from food questionnaires have linked high dietary protein to deleterious alterations in glucose metabolism [11]. Moreover, cross sectional studies have suggested that elevations in plasma branched chain amino acids are connected excess visceral adipose tissue and markers of insulin resistance [12, 13].

On the other hand, numerous studies over the past 10 years or so have demonstrated significant improvements in metabolic health with increased dietary protein intake [14–18]. In many of the cases where changes in dietary intake were implemented, it is difficult to ascertain whether the alterations were induced through dietary counseling, dietary assessment or metabolic feeding. Whereas the purpose of cross sectional studies are largely directed towards descriptive interpretation [19] and the limitations of dietary recall have been known for many years [20], conclusions drawn from these approaches still persist. In order to address the short-term impact of significant elevations in protein intake, we utilized a metabolic feeding approach that closely controlled dietary intake. In turn, we hypothesized that short-term (i.e., 4 weeks) changes in 1) elevated protein intake (EPI) that is 20% of daily calorie intake) compared to the 2) recommended level of protein intake (RPI) that is approximately 10% of daily calorie intake) would not have any measurable negative influence on hepatic and peripheral insulin sensitivity and plasma lipid profiles in older individuals with metabolic syndrome.

## Methods

### Subjects

Twelve older subjects with metabolic syndrome were recruited from the Little Rock area using local newspaper advertisements and flyers posted around the Little Rock area and the University of Arkansas for Medical Sciences (UAMS) campus (April 2013 through September 2014). Upon their first visit to the lab in the Reynolds Institute on Aging (RIOA), subjects took part in a battery of medical tests for subject eligibility, including medical history, blood count, plasma electrolytes, blood glucose concentration, and liver and renal function tests. Subjects were included if they met two of the following conditions (see Table 1): 1) plasma triglycerides (>130 mg/dl), high-density lipoprotein (HDL) (<40 mg/dl in men or < 50 ml/dl in women), blood pressure (systolic > 140 or diastolic > 90 mm Hg, or taking medication for hypertension), and fasting plasma glucose (>100 mg/dl). Subjects were excluded if they met one of following conditions: glycated hemoglobin (Hb1c) of 7.5), diabetes, lactose intolerance or dairy allergy, active malignancy within the past 6 months, gastrointestinal bypass surgery, a chronic inflammatory disease, low hematocrit or hemoglobin concentration, low platelets, concomitant use of corticosteroids, any unstable medical conditions, and use of insulin to control their blood sugar. Subjects were asked to maintain their habitual medications and to take them in a designated time (Table 1). Subjects gave written informed consent. Eligible subjects performed a dual-energy X-ray absorptiometry (QDR-4500A; Hologic, Waltham, MA) for determination of body composition (Table 1). Final analyses of the present study included twelve older adults with MS [6 subjects per group; range of age: 51–70 yrs] (Table 1) due to subject dropout (*n* = 5) and screening failures (*n* = 35): see Consolidated Standards of Reporting Trials (CONSORT) Diagram; Additional file 1: Figure S1). The sample size calculations were based on a paired *t*-test. With six subjects and a α-level of 0.05, we calculated that a paired *t*-test would have 80% power to detect effect sizes (ie., change from pre-supplementation) of 1.75 (using the Insulin Sensitivity Index) with a standard deviation of 1.6 [21]. The study was approved by the Institutional Review Board (ie., ethics approval and consent for publication committee) at UAMS. This trial is registered at https://ClinicalTrials.gov as NCT02885935.

**Table 1:** Group Characteristics and Medications

Intervention	Recommended Protein Intake		Elevated Protein Intake	
	Pre	Post	Pre	Post
Age, yrs	64.5 ± 3.0		60.2 ± 2.8	
Gender, M/F	3/3		2/4	
Total body mass, kg	108.5 ± 10.6	108.5 ± 10.5	106.0 ± 6.8	105.3 ± 6.8
Lean body mass, kg	59.0 ± 7.4	57.3 ± 6.0	58.1 ± 4.6	56.4 ± 3.8
Body mass index, kg/m^2^	37.4 ± 2.6	37.4 ± 2.6	37.6 ± 1.4	37.3 ± 1.4
Body fat (%)	41.3 ± 7.5		41.2 ± 3.4	
Total cholesterol, mg/dl	198.3 ± 43.6		188.7 ± 37.9	
Triglyceride, mg/dl	185.3 ± 60.9		171.2 ± 41.5	
HDL cholesterol, mg/dl	44.2 ± 10.1		41.5 ± 7.1	
Glucose, mg/dl	102.5 ± 23.1		99.2 ± 14.3	
Blood pressure, mm Hg				
Systolic blood pressure	143.0 ± 21.8		145.2 ± 17.2	
Diastolic blood pressure	85.0 ± 9.5		82.8 ± 5.4	
	Medications (# of subjects)			
For hypertension				
Beta blocker			2	
ACE inhibitor	1		2	
Calcium channel blocker	1		1	
Diuretic	2		5	
For lipid or type 2 diabetes				
Metformin	1		2	
Glipizide	1			
Statin	2		1	

Values are expressed as Mean ± SEM. *M/F* number of male/female subjects. There were no significance differences in any of variables of group characteristics before and after their respective dietary intervention between groups (for all; *p* > 0.10)

### Experimental protocol

After screening for subject eligibility, eligible subjects were randomly assigned by a study coordinator to one of two groups in a permuted block randomization method using a sealed envelope: the RPI or the EPI group (Additional file 2). Subjects in the RPI consumed meals consisting of 55% carbohydrate, 35% fat, and approximately 10% protein which contained 0 – 1.5 servings of dairy per day) while subjects in the EPI consumed meals consisting of 45% carbohydrate, 35% fat, and approximately 20% protein over 4 weeks. The EPI diets contained three or more servings of dairy per day compared to the RPI. Sources of the dairy were milk, yogurt, and cheese. Before and after the 4-week respective dietary intervention, subjects were studied in the RIOA for determination of insulin sensitivity and plasma lipids. The isocaloric RPI and EPI oriented diets were prepared in the Metabolic Kitchen at the RIOA by the Research Dietician. After the first metabolic studies in which participants were asked to consume their normal dietary intake, subjects in each intervention paradigm (RPI compared to EPI) consumed their respective meals for a total of 28 days (Table 2), followed by the second metabolic studies. Subjects obtained meal allotments at regular intervals from our study coordinator and were also given a dietary record and point-and-shoot digital camera. Meal consumption and percentage of meal consumption were recorded, and the meal was photographed prior to and after consumption. Subjects were instructed to return all unused or empty meal/supplement packaging and camera when they reported to the RIOA for subsequent meal allotments or for the metabolic study. These data helped the Research Dietician ascertain caloric/protein intake as well as study compliance. Prior to the first measurements and during the entire intervention periods, subjects were instructed to refrain from any significant alterations in their patterns of physical activity.

**Table 2:** Macronutrient Intake

	Recommended Protein Intake	Elevated Protein Intake
Days of diet provision	27.7 ± 0.7	28.0 ± 0.7
Daily calories intake, kcal/day	2962 ± 293	2826 ± 208
Protein		
Protein, g	77.2 ± 6.9	145.9 ± 11.6**
Protein, g/kg	0.72 ± 0.03	1.37 ± 0.02**
Protein, %	10.3 ± 0.1	20.4 ± 0.3**
EAA, g	19.8 ± 4.1	49.4 ± 10.0**
BCAA, g	9.1 ± 1.9	22.8 ± 4.6**
Leucine, g	4.1 ± 0.9	10.2 ± 2.1**
Fat		
Fat, g	117.2 ± 11.5	111.0 ± 8.2
Fat, %		
Saturated fat	10.5 ± 0.1	13.8 ± 0.1**
Monounsaturated fat	10.3 ± 0.5	9.7 ± 0.3*
Polyunsaturated fat	7.1 ± 0.1	5.1 ± 0.1**
Total fat	35.0 ± 0.1	34.9 ± 0.1
Carbohydrate		
Carbohydrate, g	412.6 ± 41.9	320.2 ± 22.8
Carbohydrate, %	54.7 ± 0.2	44.8 ± 0.3**
Fiber, g	32.9 ± 2.8	28.3 ± 1.2

Values are expressed as mean ± SEM

*Significantly different from Recommended Protein Diet, *p < 0.05, **p < 0.001

### Dual Tracer Oral Glucose Tolerance Test (DT OGTT)

**Fig. 1:**
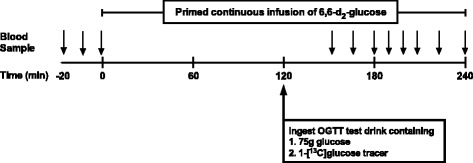
Stable isotope tracer infusion protocol

**Fig. 2:**
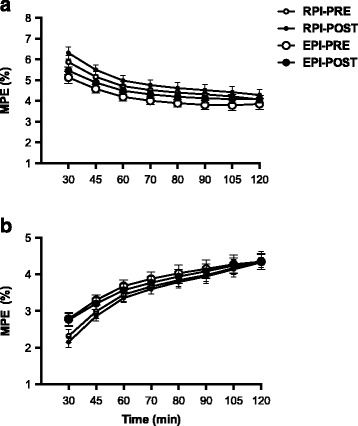
Plasma isotope tracer enrichments: **a** plasma enrichments of [6,6-^2^H_2_]glucose, which was primed continuously infused, and **b** plasma enrichments of [1-^13^C]glucose, which was ingested as a glucose bolus in which the tracer was dissolved to be enriched at ~ 5%. Values are expressed as mean ± SEM

On the 4^th^ day following 3-d of normal dietary consumption, subjects were reported to the RIOA after an overnight (after 2200) fast. Two 18- gauge catheters were placed in each lower arm, one for the infusion of stable isotope tracer and the other for blood sampling for the DT OGTT (Fig. 1). Following the collection of blood sample for determination of background isotopic enrichments, a primed, continuous [6,6-^2^H_2_]glucose infusion [prime, 82.2 umol/kg; rate, 0.78 umol•kg^−1^•min^−1^] was provided. After 2.5 h of the tracer infusion, subjects received a test drink for oral glucose tolerance test containing [1-^13^C]glucose (40 mg/kg) and 75 g of unlabeled glucose, dissolved in flavored water [22]. Over the 4.5 h period, blood samples were collected at regular intervals. The same metabolic study was replicated after completing 28 days of their respective dietary intervention. Glucose kinetics (i.e., primary outcome) including endogenous glucose rate of appearance (R_a_), exogenous glucose R_a_, and total glucose R_a_, glucose R_d_, and the metabolic clearance rate (MCR) were estimated using the Steele equation for the non-steady state [22]. Rates were calculated utilizing two adjacent two time points over the last 2 h of the DT OGTT, and the mean kinetic values were calculated from these rates. Plasma responses of glucose and insulin during the DT OGTT were determined at each of the sampling times and areas under the curves (AUCs) were calculated from these values. In addition, secondary outcome i.e., plasma concentrations of lipids in the fasted states including triglyceride, HDL, low density lipoprotein (LDL), very low density lipoprotein (VLDL), and total cholesterol were determined before and following the respective dietary interventions (Fig. 2).

### Analytical methods

Blood samples (*t* = −150, −140, and −130 min) were collected prior to the onset of the DT OGTT protocol, and serial blood samples (*t* = 30, 45, 60, 70, 80, 90, 105, and 120 min) were collected during the remainder of the study into ethylenediaminetetraacetic acid (EDTA)-containing tubes and centrifuged at 3500 rpm for 15 min at 4 °C. Plasma enrichments of glucose tracers were measured on the pentaacetate derivative with the use of gas-chromatography-mass spectrometry (models 7890A/5975; Agilent Technologies, Santa Clara, CA). Ions of mass-to-charge ratio of 331.1, 332.1, and 333.1 for glucose were monitored with chemical impact ionization and selective ion monitoring [23]. Plasma glucose concentrations were measured spectrophotometrically on a Cobas c 111 analyzer (Roche, F. Hoffman-La Roche, Basel, Switzerland). Plasma insulin concentrations were measured by using a commercially available human insulin enzyme-linked immunosorbent assay (ELISA) kit (Alpco Diagnostics). The lipid panels were determined by Labcorp (Labcorp 7777 Forest Lane, Dallas TX) using enzymatic methodology.

### Calculations

Calculations of whole body glucose kinetics in non-steady state using Steele equation [24] were performed as in our previous study [22]. In brief, plasma enrichments of glucose tracers and concentrations were curve-fitted with a 3-order polynomial model over the OGTT period in Graphpad Prism 6 for Mac (Graphpad Software, Inc. La Jolla CA). Enrichment (E) is expressed as mole percent excess (MPE): MPE is calculated as (TTR)/(1 + TTR), where TTR is tracer to tracee ratio. Appropriate corrections for skew abundance distribution and overlapping spectra were made for the glucose tracers [24]. From these calculations, total glucose R_a_ is comprised of rates of appearance of exogenous (i.e., ingested) glucose and of endogenous (i.e., hepatic glucose production and negligible renal glucose production or splanchnic glucose) glucose:

1 $$ \mathrm{Total}\;{\mathrm{R}}_{\mathrm{a}}\;\mathrm{glucose}\left({\mathrm{Total}\ \mathrm{R}}_{\mathrm{a}}\right)=\left(\mathrm{F}\hbox{--} \left(\mathrm{pV}\bullet \left({\mathrm{C}}_2+{\mathrm{C}}_1\right)/2\right)\bullet \left(\left({\mathrm{E}}_2\hbox{--} {\mathrm{E}}_1\right)/\left({\mathrm{t}}_2\hbox{--} {\mathrm{t}}_1\right)\right)\right)\ /\left({E}_2+{E}_1\right)/2 $$

2 $$ \mathrm{Glucose}\ {\mathrm{R}}_{\mathrm{d}}=\mathrm{Total}\ {\mathrm{R}}_{\mathrm{a}}\hbox{--} \mathrm{p}\mathrm{V}\bullet \left({\mathrm{C}}_2\hbox{--} {\mathrm{C}}_1\right)/\left({\mathrm{t}}_2\hbox{--} {\mathrm{t}}_1\right) $$

3 $$ \mathrm{Exogenous}\ \mathrm{glucose}\ {\mathrm{R}}_{\mathrm{a}}\left({\mathrm{R}}_{\mathrm{a}}\mathrm{Exo}\right)=\mathrm{Total}\ {\mathrm{R}}_{\mathrm{a}}\bullet \left({\mathrm{E}}_{PL}/{\mathrm{E}}_D\right) $$

4 $$ \mathrm{E}\mathrm{ndogenous}\ \mathrm{glucose}\ {\mathrm{R}}_{\mathrm{a}}=\mathrm{Total}\ {\mathrm{R}}_{\mathrm{a}}\hbox{--} \mathrm{E}\mathrm{x}\mathrm{o}\ {\mathrm{R}}_{\mathrm{a}} $$

5 $$ \mathrm{Metabolic}\ \mathrm{Clearance}\ \mathrm{Rate}\left(\mathrm{MCR}\right)={\mathrm{R}}_{\mathrm{d}}/\left(\left({\mathrm{C}}_1+{\mathrm{C}}_2\right)/2\right) $$

where F represents the infusion rate of [6,6-^2^H_2_]glucose; pV is the effective volume of distribution for glucose, for which 40 ml•kg^−1^ was used; C_1_ and C_2_ are plasma glucose concentrations at times t_1_ and t_2_, respectively, E_1_ and E_2_ are plasma enrichment of [6,6-^2^H_2_]glucose at times t_1_ and t_2_, respectively; E_*D*_ and E_*PL*_ are tracer enrichments of [1-^13^C]glucose from the test drink and plasma, respectively.

Whole-body insulin sensitivity was estimated by the Insulin Sensitivity Index (ISI) = 10,000/square root of ([fasting glucose x fasting insulin] x [mean glucose x mean insulin during OGTT]) [25].

### Statistical methods

Two-tailed independent *t*-test was used to compare changes in whole body glucose kinetics and ISI from pre- to post-intervention between RPI and EPI. Two-factor analysis of variance (ANOVA) was used to evaluate the effect of group (RPI and EPI) and intervention (before and after the respective dietary intervention) on measures of whole body glucose kinetics and AUCs of plasma glucose, insulin, and lipids. Statistical significance was declared when the *p*-value was less than 5% level. All data were analyzed using the Graphpad Prism 6 for Mac (Graphpad Software, Inc. La Jolla CA) and presented as mean ± SEM.

## Results

### Glucose kinetics and insulin sensitivity

**Fig. 3:**
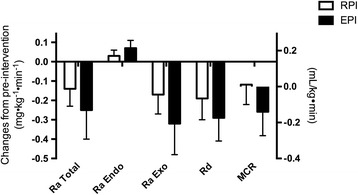
Changes in whole body glucose kinetics from baseline: Kinetic values are expressed as difference between post- and pre-interventions. Rate of appearance of total glucose (*R*_*a*_ Total), rate of appearance of endogenous glucose (*R*_*a*_ Endo), rate of appearance of exogenous glucose, rate of disappearance of glucose (*R*_*d*_), and metabolic clearance rate of glucose (MCR, *R*_*d*_ normalized to plasma glucose concentration) were determined during the OGTT before and following the 4-week diet intervention of either RPI (i.e., ~0.8 g protein/kg body weight/day) or EPI in isocaloric mixed meals intakes (i.e., ~1.4 g protein/kg body weight/day). Values are expressed as mean ± SEM

Whole body glucose kinetics are presented as absolute (Table 3) and changes from pre- to post-intervention (ml/kg/min for MCR and mg/kg body weight/min for the other variables) (Fig. 3). For all these kinetic variables, we did not find any significant differences between RPI and EPI.

**Table 3:** Whole body glucose kinetics during the oral glucose tolerance test over 4-week dietary intervention

	Recommended Protein Intake		Elevated Protein Intake		*p*-value		
	Pre	Post	Pre	Post	Time	Group	T × G
*R*_*a*_ Total, mg/kg/min	3.39 ± 0.13	3.25 ± 0.17	3.75 ± 0.18	3.50 ± 0.19	0.045^a^	0.192	0.533
*R*_*a*_ Endo, mg/kg/min	1.20 ± 0.09	1.22 ± 0.11	1.08 ± 0.09	1.15 ± 0.10	0.062	0.501	0.369
*R*_*a*_ Exo, mg/kg/min	2.19 ± 0.15	2.02 ± 0.22	2.67 ± 0.25	2.35 ± 0.20	0.027^a^	0.174	0.442
*R*_*d,*_ mg/kg/min	3.25 ± 0.17	3.06 ± 0.19	3.71 ± 0.18	3.42 ± 0.14	0.015^a^	0.110	0.544
MCR, ml/kg/min	1.67 ± 0.16	1.68 ± 0.24	1.97 ± 0.36	1.83 ± 0.29	0.486	0.558	0.391

Values are expressed as mean ± SEM

*R*_*a*_ *Total* Rate of appearance of total glucose, *R*_*a*_ *Endo* rate of appearance of endogenous glucose, *R*_*a*_ *Exo* rate of appearance of exogenous glucose, *R*_*d*_ rate of disappearance of glucose, *MCR* metabolic clearance rate of glucose (*R*_*d*_ normalized to plasma glucose concentration), *T × G* time-group interaction

^a^There were significant effects on time (i.e., post- vs. pre-intervention). However, post hoc *t*-test analyses revealed no significant changes from pre- to post-intervention (for all, *p* > 0.235)

### Plasma glucose and insulin responses

**Fig. 4:**
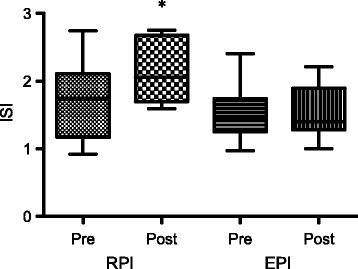
Insulin sensitivity index: Pre- and post-intervention values in RPI and EPI. *Represents significant increase from pre-intervention

For plasma glucose AUC responses, there were no significant effects for a group-by-intervention interaction (*p* = 0.573), for group (*p* = 0.756) and for intervention (*p* = 0.620) for glucose AUC (Table 4). For plasma insulin AUC responses, there were no significant effects for a group-by-intervention interaction (*p* = 0.7892), for group (*p* = 0.187), and for intervention (*p* = 0.080) (Table 4). However, ISI increased significantly in RPI (*P* = 0.02), but did not change in EPI (*P* = 0.90) (Fig. 4).

**Table 4:** Plasma glucose and insulin responses during the oral glucose tolerance test and fasting lipids over 4-week dietary intervention

	Recommended Protein Intake		Elevated Protein Intake	
	Pre	Post	Pre	Post
Glucose AUC	22504 ± 2062	21745 ± 2789	23286 ± 2615	23335 ± 3097
Insulin AUC	10022 ± 2122	8314 ± 1899	14831 ± 3026	12561 ± 2300
Total cholesterol	184.8 ± 12.9	176.7 ± 21.6	179.5 ± 13.2	170.2 ± 10.4
Triglyceride	154.5 ± 19.3	175.3 ± 18.9	188.5 ± 18.2	192.0 ± 29.3
HDL cholesterol	36.8 ± 3.3	34.3 ± 3.6	35.5 ± 8.3	34.8 ± 9.8
LDL cholesterol	116.8 ± 11.5	108.3 ± 17.3	106.3 ± 12.9	97.7 ± 9.7
VLDL cholesterol	31.2 ± 3.9	35.3 ± 3.9	37.7 ± 8.7	38.5 ± 5.8

Values are expressed as mean ± SEM (mg/dl)

*AUC* area under the curve, *HDL* cholesterol, high-density lipoprotein cholesterol, *LDL* cholesterol, low-density lipoprotein cholesterol, *VLDL* cholesterol, Very-low density lipoprotein cholesterol

### Fasting plasma lipids

There were no significant group-by-intervention interactions, group effects, and intervention effects of total, HDL, LDL, and VLDL cholesterol and triglyceride (for all, *p* > 0.05) (Table 4).

## Discussion

Consistent with our hypothesis regarding the short-term influence on glucose metabolism, we did not observe any adverse changes in hepatic and peripheral insulin sensitivity (as evaluated by glucose R_a_, glucose R_d_ and MCR during the DT OGTT) and/or plasma lipid profiles in older individuals with metabolic syndrome following either the RPI (i.e., 0.72 g protein/kg body weight/day) or EPI (i.e., 1.37 g protein/kg body weight/day) after 4 weeks of respective dietary intervention. More specifically, glucose R_a_, glucose R_d_ and MCR during the DT OGTT were not different between groups and there was no difference in glucose and/or insulin AUC following the respective interventions. ISI was increased in the RPI and did not change in EPI. This difference was largely influenced by outliers in two participants in each insulin data set that were reduced in RPI, and yet increased in EPI. There were no differences in fasting blood lipids between RPI and EPI. Unlike evidence from epidemiological studies that suggest a negative effect of increased dietary protein on glucose metabolism [9], direct short-term elevation of dietary protein intake (i.e., EPI) from dairy products does not have a negative effect on insulin resistance and/or lipid parameters in older adults with metabolic syndrome.

It has been suggested that high protein intake induces insulin resistance via leucine-mediated activation of the mechanistic target of rapamycin (mTOR) [26]. This hypothesis is largely based upon a positive correlation between tissue or plasma concentrations of branched-chain amino acids (BCAA) and insulin resistance in obese individuals [27], and based upon data showing impaired insulin-mediated glucose uptake with in vitro leucine treatment via this mechanism [28, 29]. This postulation was further strengthened by the observations in humans that intravenous amino acid infusion reduced glucose uptake during hyperinsulinemic euglycemic clamp [30, 31], although the role for leucine in the pathogenesis of insulin resistance has been challenged [32]. However, there are several issues to consider. It was not established whether the elevated BCAA concentrations in obese insulin resistant individuals were the cause or an effect of insulin resistance. Second, reduced glucose uptake as a result of amino acid infusion during the clamp (a non-physiological condition) may not reflect the glucose metabolism in the physiological circumstance of mixed meal intake with varying protein or BCAA amount. It is also possible that acute physiological responses may not reflect chronic responses to high protein or BCAAs, necessitating interventional studies and chronic response determination.

Despite significant interests in the effects of high protein diets on glucose metabolism, the study of these diets in humans under weight-stable conditions using controlled metabolic feeding has been surprisingly scarce [33–35]. In the present study, we observed no impairment in glucose disposal during a physiological condition (i.e., OGTT) following 4 weeks of EPI containing more than 2-fold higher leucine or BCAA contents than RPI. Consistent with our findings, Shiu et al. found that consumption of a high protein diet for 4 weeks did not alter plasma glucose/insulin concentrations or insulin sensitivity as assessed by an intravenous glucose tolerance test [35]. In longer-term weight-stable studies where participants served as their own controls, and wash-out periods in between three different types of diets (including the Dietary Approaches to Stop Hypertension (DASH), protein rich diet or unsaturated fat rich diets), insulin sensitivity as assessed by the quantitative insulin sensitivity check index (QUICKI) and homeostasis model assessment of insulin resistance (HOMA-IR) was not different [34]. Lastly, in studies comparing four different types of diet (Control, High cereal fiber, High Protein, and High Cereal Fiber/High Protein), the High Protein diet had no influence on markers of insulin sensitivity (i.e., QUICKI). In individuals with more profound insulin resistance, Gannon et al., demonstrated that higher protein intake (30% protein vs. 15% protein in total energy of the daily meals) for 5 weeks in type 2 diabetic patients resulted in a 40% reduction in the mean 24-h integrated glucose AUC (mean age: 61y, range: 39 – 79y) [36]. Glycated hemoglobin also decreased significantly with 5 weeks of higher protein intake in this study providing additional evidence to the importance of this strategy in individuals with type 2 diabetes. On other hand, in studies where individuals had yet to be classified with type 2 diabetes [33–35], consumption of increased dietary protein was not sufficient to improve insulin sensitivity even when measured with methods that provided enhanced specificity [35].

Given the epidemiological evidence showing a significant inverse relation between dairy product intake and metabolic syndrome [37], improvements in peripheral insulin sensitivity with EPI can be expected, as EPI contained > 2-fold higher dairy products, compared to RPI. It is possible that to observe beneficial effects on peripheral insulin sensitivity, a higher relative protein intake (i.e., 30% of overall energy intake) is required. For example, the higher relative protein intake of Gannon study was not only 1/3 greater than ours, but was also lower in dietary carbohydrate. The combination of higher protein and lower carbohydrate intake may be required to elicit alterations in glucose metabolism. Alternatively, it may be likely that a longer time is required to realize the beneficial effects of increasing dairy protein intake in mixed meals with respect to insulin sensitivity.

As secondary outcomes in the present study, we determined lipid panels in the fasted state before and after the respective interventions. In many cases, studies that have demonstrated the effectiveness of increased dietary protein intake on improvements in blood lipids were also associated with weight loss [15, 38, 39]. Meta-analysis of high protein/weight loss studies has confirmed their preferential efficacy on the reduction of triglycerides in particular [40]. In the case of a hypercaloric diet, increased protein intake is linked to a trend (i.e., *p* = 0.07) towards reduced triglycerides [7]. In our study under conditions of weight balance and isocaloric dietary intake, we found no improvements in any of the lipid panels (Table 4). Consistent with our findings, Chiu et al. found no improvement in triglyceride and total-, LDL-, or HDL-cholesterol after 4 weeks of either 20% (as in the present study) or 30% of protein with either low or high saturated fat intake without weight loss in overweight and obese adults [35]. Therefore, an elevation in dietary protein intake without weight loss seems to foster stable lipid parameters in individuals with the characteristics of metabolic syndrome.

A potential limitation of the present study is that we did not quantify habitual protein intake or dietary patterns of the subjects prior to study initiation. Thus, it is possible that subjects in the EPI group may not have consumed much more protein than their usual protein intake. In retrospect, study design may have been better served with a longer dietary run-in period to reduce the potential influences of subjects’ previous dietary pattern [41]. If we assume that most individuals were eating the average American protein intake by NHANES (i.e., 1.1 g/kg/day; we had no vegetarians in the study), then the variance from habitual protein intake to that consumed between groups (0.72 g/kg/d vs. 1.37 g/kg/d) should only serve to magnify the response to changes in protein intake in this population, if indeed one exists. We have illustrated that elevation of dietary protein intake using dairy products does not have a negative influence on insulin sensitivity. While some studies have linked increased consumption of red meat to the development of insulin resistance [42], recent data from longitudinal feeding studies dispute this assertion [43]. These findings indicate that reasonable elevations in protein intake do not alter glucose kinetics in subjects with metabolic syndrome, and also highlight the empirical nature of nature of epidemiological research [44].

## Conclusions

In the present study, we found that 4 weeks of higher protein intake (i.e., EPI) containing a significant amount of dairy products and BCAAs did not improve nor worsen glucose metabolism as measured by isotopically measured glucose kinetics, and lipid parameters in individuals with the clinical characteristics of metabolic syndrome. Unfortunately, interpretation of the ISI was complicated by two outliers in each insulin data set for RPI and EPI. Future studies with a longer intervention period should be performed to ascertain whether increasing amount of “high quality” protein intake containing correspondingly high BCAA have positive or negative impact on the modulation of glucose and lipid parameters.

## Additional files

Additional file 1: Figure S1. Consort flow chart. (DOC 49 kb)

Additional file 2: Study protocol. (PDF 1191 kb)

## Abbreviations

RPI: Recommended protein intake
EPI: Elevated protein intake
DT OGTT: Dual Tracer Glucose Tolerance Test
Glucose R_a_: Glucose rate of appearance
Glucose R_d_: Glucose Rate of Disappearance
MCR: Metabolic Clearance Rate of Glucose/Glucose Concentration
BCAA: Branched chain amino acids
RDA: Recommended Daily Allowance
UAMS: University of Arkansas for Medical Sciences
RIOA: Reynolds Institute on Aging
HDL: High-density lipoprotein
Hb1c: Glycated hemoglobin
CONSORT: Consolidated Standards of Reporting Trials
AUCs: Area under the curves
VLDL: Very low density lipoprotein
ELISA: Enzyme-linked immunosorbent assay
MPE: Mole percent excess
TTR: Tracer to tracee ratio
F: Infusion rate of [6,6-^2^H_2_]glucose
pV: Effective volume of distribution for glucose
C_1_ and C_2_: Plasma glucose concentrations at times t_1_ and t_2_
E_1_ and E_2_: Plasma enrichment of [6,6-^2^H_2_]glucose at times t_1_ and t_2_
E_*D*_ and E_*PL*_: Tracer enrichments of [1-^13^C]glucose from the test drink and plasma
mTOR: Mechanistic target of rapamycin
ANOVA: Analysis of variance
DASH: Dietary Approaches to Stop Hypertension
QUICKI: Quantitative insulin sensitivity check index
HOMA-IR: Homeostasis model assessment of insulin resistance
