# Supplementary material for: Short term elevation in dietary protein intake does not worsen insulin resistance or lipids in older adults with metabolic syndrome: a randomized-controlled trial
Source: BMC Nutr. 2017 Apr 17;3:33. doi: 10.1186/s40795-017-0152-4 (PMC5510665; doi:10.1186/s40795-017-0152-4)
Supplement: Supplementary file 1 — Consort flow chart. (DOC 49 kb) [file 40795_2017_152_MOESM1_ESM.doc]

**
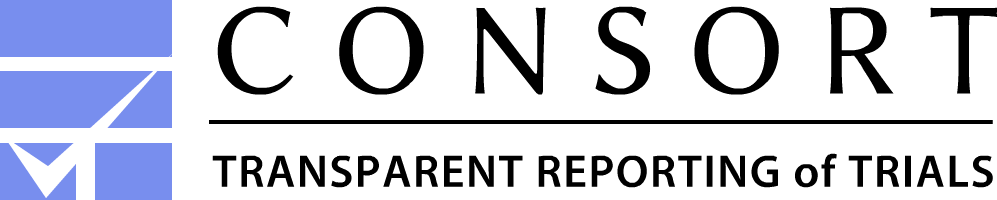
**

**CONSORT 2010 Flow Diagram**

**Allocation**

**Analysis**

**Follow-Up**

**Enrollment**

Assessed for eligibility (n= 47)

Excluded (n= 35 )

♦  Not meeting inclusion criteria (n= 30 )

♦  Declined to participate (n= 5 )

♦  Other reasons (n= 0 )

Analysed (n= 6)
♦ Excluded from analysis (give reasons) (n=0)

Lost to follow-up (give reasons) (n= 0)

Discontinued intervention (give reasons) (n=0)

Allocated to ‘RPI’ intervention (n= 6)

♦ Received allocated intervention (n= 6)

♦ Did not receive allocated intervention (n=0)

Lost to follow-up (give reasons) (n= 0)

Discontinued intervention (give reasons) (n=0)

Allocated to ‘EPI’ intervention (n= 6)

♦ Received allocated intervention (n= 6 )

♦ Did not receive allocated intervention (n= 0 )

Analysed (n=6)
♦ Excluded from analysis (give reasons) (n=0)

Randomized (n= 47)
