# Supplementary material for: Short term elevation in dietary protein intake does not worsen insulin resistance or lipids in older adults with metabolic syndrome: a randomized-controlled trial
Source: BMC Nutr. 2017 Apr 17;3:33. doi: 10.1186/s40795-017-0152-4 (PMC5510665; doi:10.1186/s40795-017-0152-4)
Supplement: Supplementary file 2 — Study protocol. (PDF 1191 kb) [file 40795_2017_152_MOESM2_ESM.pdf]

Dairy Macronutrient Effects on the Metabolic Syndrome

Principal Investigators:

Arny Ferrando, PhD., Professor, Geriatrics
Center for Translational Research in Aging & Longevity,
The University of Arkansas for Medical Sciences
4301 West Markham St., Slot 806
Little Rock, AR 72205
Office Telephone: 501-526-5711
EMAIL address: aferrando@uams.edu

Robert H. Coker III, PhD, Associate Professor of Biology and Wildlife
Center for Alaska Native Health Research
Institute for Arctic Biology
311 Irving I, Box 757000
University of Alaska Fairbanks
Fairbanks, AK 99775-7000
EMAIL address: rcoker@alaska.edu

Sub-Investigators:
Gohar Azhar, MD
Il-Young Kim, PhD
Scott Schutzler, RN
Robert R. Wolfe, PhD
Center for Translational Research in Aging &
Longevity, The University of Arkansas for Medical
Sciences 4301 West Markham St., Slot 806
Little Rock, AR 72205

Funding Support:
Dairy Research Institute

Contents

1.0 Schema **4**
2.0 Table of Events **6**
3.0 Summary of Project **7**
4.0 Hypothesis **7**
4.1 Specific Aims 7
5.0 Background Information **8**
5.1 Review of Literature 8
5.2 Preliminary Data 11
6.0 Trial Design **12**
6.1 Inclusion Criteria 13
6.2 Exclusion Criteria 13
6.3 Randomization 13
6.4 Study Interventions 14
6.5 Study Procedures 14
6.5.1 Stable isotope studies for determination of glucose kinetics 14
6.5.2 Whole body DEXA scan 15
6.5.3 Blood sampling 15
6.6 Subject Compensation 16
6.7 Blinding 16
6.8 Subject compliance 16
6.9 Subject Recruitment 17
7.0 Analytical Methods **17**
7.1 Blood plasma analyses 17
7.2 Statistical Analysis 18
7.3 Power and Sample Size 18
7.4 Interim Analyses 18
7.5 Data Storage 19
7.6 Sample Storage 19
8.0 Safety Reporting **19**
8.1 Definitions 19
8.2 (Serious) Adverse Events handling 20

8.3 (S)AE reporting 20
8.4 Follow-up of (S)AEs 20
8.5 Risks/Benefits 20
8.5.1 Peripheral catheter placement: 21
8.5.3 Blood sampling: 21
8.5.4 Stable isotope infusion: 21
8.5.5 Stable Isotope ingestion: 22
8.6 Informed Consent Process 22
9.0 Use of Data 23
10.0 References 24
11.0 Appendices 29
11.1 Medical History and Physical Examination 29
11.2 Stable Isotope Infusion SOP 30
11.3 Advertisement 33
11.4 Introductory Letter to Potential Subjects 34
11.5 Diet Diary 36

1.0 Schema

RECRUITMENT

Advertisement or Telephone Response

Use Research Match.org via TRI @ UAMS

UAMS Data Warehouse search

VISIT 1

Screening Visit @ Donald W. Reynolds Institute on Aging

Sign Consent Form

Anthropometrics and vital signs

Medical History and Physical Exam

Blood sample for eligibility (HbA1c, lipid panel)

Provide 3 days of diet diaries, provide instructionsDEXA scan for lean body mass

With their doctor's permission, subjects hold all diabetes meds from now through Visit 10

VISIT 2 (approx. 1-2 weeks after V. 1)

Arrive to RIOA fasted overnight

Perform 4-hour stable isotope/glucose tolerance test

Blood sampling

Meet with study dietician

Return diet diaries or mail to dietician

VISIT 3 (approx. 2-4 days after V. 2)

@ RIOA

Retrieve meals for 2-4 days

Weigh subject

Retrieve meal photos from camera

VISIT 4 (approx. 2-4 days after V. 3)

@ RIOA

Retrieve meals for 2-4 days

Weigh subject

Retrieve meal photos from camera

VISIT 5 (approx. 2-4 days after V. 4)

@ RIOA

Retrieve meals for 2-4 days

Weigh subject

Retrieve meal photos from camera

VISIT 6 (approx. 2-4 days after V. 5)

@ RIOA

Retrieve meals for 2-4 days

Weigh subject

Retrieve meal photos from camera

VISIT 7 (approx. 2-4 days after V. 6)

@ RIOA

Retrieve meals for 2-4 days

Weigh subject

Retrieve meal photos from camera

VISIT 8 (approx. 2-4 days after V. 7)

@ RIOA

Retrieve meals for 2-4 days

Weigh subject

Retrieve meal photos from camera

VISIT 9 (approx. 2-4 days after V. 8)

@ RIOA

Retrieve meals for 2-4 days

Weigh subject

Retrieve meal photos from camera

VISIT 10 (approx. 2-4 days after V. 9)

@ RIOA

Retrieve meals for 2-4 days

Weigh subject

Retrieve meal photos from camera

VISIT 11

Arrive to RIOA fasted overnight

Perform 4-hour stable isotope/glucose tolerance test

Vital signs

Blood sampling

Weigh subject

Retrieve camera

DEXA scan for lean body mass

Provide light meal

2.0 Table of Events

Procedure	Visit 1	Visit 2	Visits 3-10	Visit 11
**Informed consent process**	X			
**Anthropometrics**	X			
**Vital signs**	X	X		X
**Medical history**	X			
**Physical exam**	X			
**Blood sampling**	X[1]	X[2]		X[2]
**Stable isotope infusion/ingestion**		X[3]		X[3]
**OGTT**		X[4]		X[4]
**Meet with dietician**		X		
**Diet Diaries**	X			
**DEXA scan**	X[5]			X[5]
**Retrieve study meals**			X	
**Photograph study meals**			X	
**Weigh subject**	X		X	X

[1]For eligibility criteria: HbA1c, lipid panel at LabCorp.
[2]Fasted samples for study endpoints: glucose, insulin, stable isotopes, C-peptide, plasma lipids, C-reactive protein, TNF-alpha, and IL-6. Visit 11 will also have HbA1c testing.
[3]Infusion of 6-6-2H2 glucose for 4 hours and ingestion of 1-13C glucose isotope one time.
[4]Ingestion of 75g oral glucose tolerance test solution.
[5]DEXA scan at RIOA 3rd floor for whole body analysis of body composition.

3.0 Summary of Project

We will investigate changes in insulin sensitivity before and after 4 weeks of dietary intervention and control in subjects with metabolic syndrome. Two groups of subjects will be studied before and after a weight maintenance diet. Group 1 will be fed a common American diet with a macronutrient distribution of 10% protein, 55% carbohydrates, and 35% fat. Group 2 will consume a higher protein diet (20%; 1.5 g/kg/d of protein), the proposed optimal intake for an older population[1], with dairy protein as the predominant source of protein. Carbohydrate intake will be lower in Group 2 (45%), with fat intake (largely derived from dairy sources) similar between groups. Glucose utilization, endogenous glucose production, and insulin secretion will be determined during an oral glucose tolerance test (OGTT) with a novel double-tracer approach. Our secondary aim will be to determine the effect of a diet high in dairy consumption on inflammatory cytokines and blood lipid profiles. We will measure blood lipids and pro-inflammatory cytokines before and after dietary intervention in each group.

4.0 Hypothesis

Higher dairy protein and lower carbohydrate intake will result in a better metabolic profile versus a diet which is lower in dairy/protein intake and higher in carbohydrate.

4.1 Specific Aims

Our **first specific aim** will investigate glucose utilization and endogenous glucose production during an oral glucose tolerance test (OGTT) with a novel double-tracer approach. We will determine insulin sensitivity by measuring glucose disposal and glucose production, as well as insulin secretion.

Our **second specific aim** will be to compare the effects of the two dietary interventions on certain risk factors for metabolic disease, namely blood lipid and pro-inflammatory cytokine profiles. We will measure blood lipids (total cholesterol, HDL, LDL, and triglycerides) and systemic indices of inflammation, such as C-reactive protein (CRP), TNF-α, and IL-6 before and after dietary intervention in each group.

5.0 Background Information

5.1 Review of Literature

Dairy Fat in a Better Light

Recent reviews of epidemiological data have demonstrated that individuals with a greater consumption of dairy foods are less likely to develop cardiovascular disease (CVD)[2]. This inverse correlation between dairy consumption and CVD may seem counterintuitive, given the accepted evidence which links consumption of saturated fat to increased CVD risk factors. While the stronger association between dairy intake and reduced CVD risks exists with low-fat products[2], there is still evidence to suggest that all saturated fat is not equally bad. For example, Tholstrup et al.[3] conducted a 3 wk dietary study to investigate the effects of fat intake from whole milk, butter, or cheese on blood lipids and postprandial glucose/insulin response. The authors found that a diet high in butter resulted in significantly greater total and LDL cholesterol concentrations than a diet high in cheese[3]. Overall, examination of most observational studies reveals *no* association between the intake of dairy products and the *increased* risk of CVD, coronary heart disease, or stroke, regardless of milk fat levels[4]. Rather, closer examination of the epidemiological data indicates the advantages of higher dairy intake on the non-lipid related CVD risk factors.

Dairy and Reduction of CVD Risk Factors

The association between hypertension and CVD is well-established. A number of dietary strategies are often employed to reduce blood pressure, including the restriction of dietary sodium and alcohol intake. However, dairy consumption as a preventative measure for hypertension has been derived from substantial evidence from the NHANES I study. In the examination of over 10,000 individuals, it was noted that lower consumption of milk products was associated with a higher incidence of hypertension[5]. Further evidence lies in the prospective examination of almost 29,000 women over 45 yrs of age who were followed over a 10 yr period. The findings indicate that the highest intake of low-fat dairy products was associated with a significant 11% reduction in the risk of developing hypertension[6]. The most acknowledged intervention study on the association between dairy foods and blood pressure is the multicenter DASH (Dietary Approach to Stop Hypertension) study. The DASH diet, which was rich in fruits and vegetables, as well as low-fat dairy products, reduced systolic and diastolic pressure by 5mm Hg and 3 mm Hg, respectively[7]. While this reduction may not seem clinically

relevant, it has been estimated that population-wide changes of this magnitude would reduce the incidence of heart disease by approximately 15% and stroke by approximately 27%[8].

Dairy foods have also been shown to reduce inflammatory and oxidative stress, two key etiological factors in the development of atherosclerosis and CVD. A recent study in a transgenic rat model of diet-induced obesity fed a high dairy diet demonstrated an adipose tissue reduction in the inflammatory cytokines IL-6 and TNFα[9]. Twenty-four week feeding of a high-dairy eucaloric and hypocaloric diet in overweight men and women significantly reduced plasma CRP and adiponectin concentrations[9]. The clinically significant reductions in each gender suggest that high dairy consumption suppresses adipose tissue oxidative and inflammatory stress that accompanies obesity. These data also suggest that the alterations in CRP and adiponectin are independent of changes in body weight.

The incidence of type 2 diabetes (DM) is increasing at an alarming rate, and is itself an important risk factor for CVD. In addition to its association with a dysregulation of glucose-insulin homeostasis, DM is a dyslipidemic and pro-inflammatory state, each constituting singular and combined risk factors for CVD[10]. Lifestyle changes and dietary modifications are the primary recommendations for the prevention and treatment of DM. In this regard, dairy foods exhibit some important anti-diabetic properties. Observational studies have consistently demonstrated an association between dairy intake and DM. Specifically, the highest vitamin D and calcium intake has been associated with an 18% reduction in DM, while general dairy intake reduces the risk of DM by 14%[11]. The association of vitamin D and calcium intake to the reduced risk of DM is more pronounced in populations with existing glucose intolerance[11].

For this reason, it is relevant to examine the relationship of dairy intake and the metabolic syndrome. Dairy consumption has been shown to be inversely associated with several aspects of the metabolic syndrome. NHANES data derived from over 4500 people with metabolic syndrome revealed a significant inverse correlation between the intake of whole milk, yogurt, calcium, and metabolic disorders[12]. The authors noted that ethnic differences related to the incidence of metabolic syndrome were partly explained by variations in dairy intake and related nutrients[12]. In the CARDIA study involving 3157 adults between the ages of 18 and 30 yrs, consumption of ≥ 5 servings/d of dairy products reduced the risk of developing the metabolic syndrome by 70% over those consuming < 1.5 serving/d over a 10 yr period[13]. Further, the association between dairy intake and metabolic syndrome was also demonstrated in the Women's Health Study[14]. Based upon these data, we propose to study individuals with metabolic syndrome for two primary reasons. First, it precludes complications due to the insulin-sensitizing agents and medications that accompany overt DM. Second, based upon the existing

data and our experience, we believe that this group is likely to realize changes in glucose disposal with careful dietary control.

The Protein Component and its Benefits

Many of the individual components of dairy foods, such as milkfat, vitamin D, calcium, magnesium, potassium, and whey protein have demonstrated benefits towards health promotion[15]. However, our extensive work with essential amino acids (EAA) and their beneficial effects on muscle[16], blood lipids, and insulin sensitivity (see Preliminary Data) enable us to focus upon the effects of dairy proteins, which contain a high proportion of EAA. While the exact mechanisms explaining the demonstrated effects of EAA on blood lipids and insulin sensitivity have yet to be elucidated, existing research on milk proteins provide important insight. Of the two primary milk proteins, casein and whey, there is substantial evidence as to the benefits of whey protein on several aspects of the metabolic syndrome. Whey protein has been associated with improved lipid profiles[17], blood pressure in hypertensive individuals[18]), and insulin sensitivity[17, 19]. Improved lipid profiles with whey protein may be due to the inhibition of angiotension-converting enzyme (ACE) and the subsequent inhibition of angiotensin II hormone[20]. Angiotensin II has a well-established role in blood pressure management[18] but is also involved in adipocyte lipogenesis[21]. Thus, the inhibition of ACE leads to a reduction in endogenous fat production[22], a decrease in plasma triglycerides[23] , and the transport of the low density cholesterol fraction[17]. In a clinical trial, whey consumption reduced TG by 13% after 6 week of supplementation[17], which is consistent with our findings after 4 week of EAA + whey supplementation (Preliminary Data). Whey is a rapidly digested protein resulting in a higher peripheral amino acid concentrations[24] and a concomitant stimulation of the insulin response[25]. Blood glucose was significantly reduced after whey protein consumption[25] and twice daily supplementation of meals with whey protein for 12 weeks resulted in a significant decrease in fasting insulin[17]. These results are consistent with our data demonstrating the effects of higher EAA + whey consumption/supplementation on blood lipids and glucose control/insulin sensitivity. Thus, we propose to examine the effects of higher diary protein consumption on insulin sensitivity and blood lipids, and compare these parameters to a common American diet with lower dairy protein and higher carbohydrate consumption.

The Real CVD Culprit may be…

The American diet has evolved to a greater consumption of carbohydrates, and in particular, carbohydrates with little nutritional value and high glycemic index (GI). Chronic

feeding of high GI carbohydrates to rats actually induces the metabolic syndrome, with increased visceral adiposity and reduced lean mass, increased intramuscular lipid, impaired glucose and insulin tolerance, and increased infiltration of inflammatory cells in the heart and liver[26]. Others have also demonstrated that rats fed a high carbohydrate, high-fat diet exhibit impaired glucose tolerance[27, 28], in great part by decreasing insulin-stimulated muscle glucose transport[28]. In rats, a high carbohydrate, high fat diet is also related to higher adiposity[29], cardiovascular remodeling, impaired ventricular function, and increased liver disease with concomitant increases in protein levels of NF-κB[27].

While epidemiological data in humans indicates similar relationships, prospective studies which alter the type or amount of carbohydrate intake demonstrate adverse effects on CVD risk factors. Recently, the Canadian Trial of Carbohydrates in Diabetes investigated postprandial changes in glucose and triglycerides in response to high GI carbohydrates. Higher GI carbohydrate intake was associated with increased postprandial glucose and triglyceride concentrations[30]. An early investigation demonstrated the effects of increasing fat intake in non-diabetic subjects. Controlled metabolic feeding was conducted for 14d whereby fat intake was increased from 15 to 50% of the caloric intake. The higher fat diet was associated with decreased oral glucose tolerance and increased cholesterol concentrations[31]. The decline in glucose tolerance was associated with reduced glucose disposal and insulin secretion[31]. Thus, the body of animal and human research indicates a clear relationship between the intakes of lipogenic precursors, whether it be high GI carbohydrate or high saturated fat, and primary metabolic alterations associated with the metabolic syndrome. Existing evidence indicates that this dietary pattern is becoming more prevalent among Americans. Thus, we propose that by altering this pattern to include a higher consumption of dairy products, with their concomitant quality protein and fat distribution, an improvement in CVD risk factors will be realized.

5.2 Preliminary Data

Dairy proteins (casein and whey) are complete proteins with high biological value, owing to the completeness of amino acid profile, in particular, essential amino acid content. Approximately 40-45% of milk proteins consist of EAA, of which just over 10% is leucine, an important regulator of muscle protein metabolism[32, 33]. While we have repeatedly demonstrated the effects of EAA on muscle protein anabolism[16], recent work from our laboratory has demonstrated the effects of an EAA-enhanced whey formula in decreasing risk factors associated with CVD . In a pilot study, we administered a nutritional formula containing approximately 9g EAA's derived from both free amino acids and whey protein. Nine middle-

aged individuals with mild hypertriglyceridemia were given this formula TID for 4 weeks. To put this intake into dietary perspective, the leucine intake was equivalent to approximately 40g of dairy protein, or approximately 3 servings of milk per day. This dairy protein intake is quite reasonable for a 75 kg man consuming 1.5g/kg/d of protein, as we propose, and has been linked to a reduced incidence of metabolic syndrome[13]. The results of our study demonstrated that TID consumption of this amino acid profile resulted in a significant decrease in triglycerides, total cholesterol, VLDL, and LDL (Figure 1A). In addition, formula administration resulted in a significant increase in insulin sensitivity (Figure 1B) as determined by oral glucose tolerance test (OGTT). These data indicate that EAA/leucine consumption comparable to a higher dairy intake can reduce several important risk factors for CVD. Further, the data demonstrate the ability to improve insulin sensitivity in a population exhibiting conditions of the metabolic syndrome within the proposed dietary intervention period. These data shed new light on the potential benefits of higher EAA/leucine ingestion, and offer a potential explanation for the observed benefits of higher dairy consumption. Given the favorable amino acid profile of dairy protein, including leucine, our data offer a feasible hypothesis for the observed relationship between dairy intake and lower CVD risks. We will explore this hypothesis further and anticipate that the favorable content of EAA/leucine in dairy products will serve to decrease blood lipids and enhance insulin sensitivity in subjects with metabolic syndrome.

6.0 Trial Design

Our goal is to study up to 12 subjects in each of two groups between the ages of 45-75 years from Central Arkansas with metabolic syndrome utilizing a modified definition from the International Diabetes

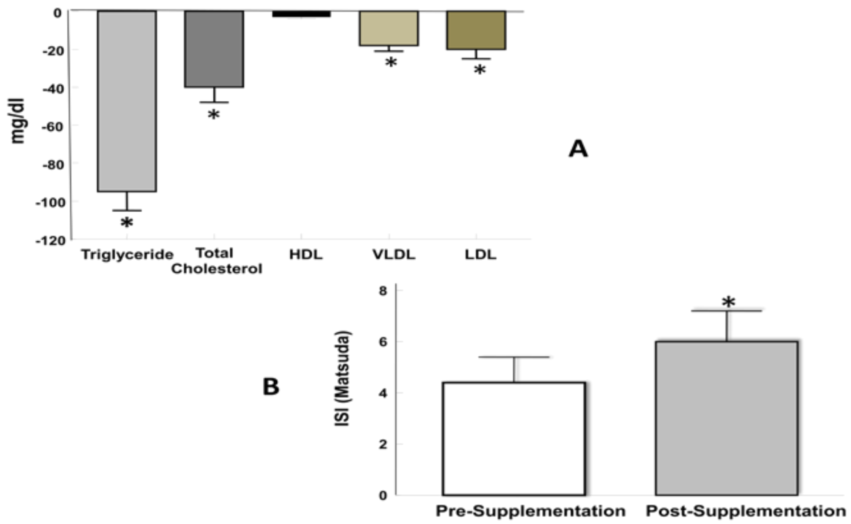

Figure 1. Panel A represents the reduction in lipid parameters following BID supplementation with a nutritional formula of essential amino acids and whey protein in subjects with mild hypertriglyceridemia. Panel B denotes the concomitant improvement [illegible]

Foundation (IDF). Up to 50 subjects will be enrolled to allow for screen failures and incomplete subjects.

6.1 Inclusion Criteria

- Men and women, ages 45-75 years AND
- BMI greater than 30 kg/m^2 AND
- Two of the following conditions:
    - o Plasma triglycerides > 130 mg/dl
    - o HDL < 40 mg/dl in men or 50 mg/dl in women
    - o elevated screening blood pressure (systolic > 140 or diastolic >90 mm Hg) or taking medication for hypertension
    - o fasting plasma glucose > 100mg/dl

6.2 Exclusion Criteria

- Hemoglobin A1c > 7.5
- History of malignancy in the 6 months prior to enrollment
- History of lactose intolerance or dairy allergy
- History of gastrointestinal bypass surgery (Lapband, etc)
- History of a chronic inflammatory condition or disease (Lupus, HIV/AIDS, etc)
- Subjects who do not or will not eat animal proteins
- Subjects who cannot refrain from consuming protein or amino acid supplements during their participation in this study
- Subjects who use insulin to control their blood sugar
- Subjects whose physician will not allow suspension of oral diabetes medications for the duration of the study (~5 weeks)
- Concomitant use of corticosteroids (ingestion, injection or transdermal)
- Any other disease or condition that would place the subject at increased risk of harm if they were to participate, at the discretion of the study physician

6.3 Randomization

Randomization order will be predetermined by coin flip method. Subjects who are found eligible at Visit 1 will be randomized to one of two groups: group 1 or group 2.

6.4 Study Interventions

Group 1 will consume 28 (±2) days of a macronutrient intake consistent with Center for Disease Control and NHANES data regarding the average American diet[35]. Macronutrient intake will consist of 55% carbohydrate, 35% fat, and approximately 10% protein.
Group 2 will consume 28 (±2) days of a diet containing a greater amount of protein (1.5 g/kg/d of protein; 20%), weighted towards dairy protein with its accompanying fat content (both low and normal fat products; 35% fat), with the remainder of caloric requirement being carbohydrate (45%). This study design enables several important investigations. First, we will determine the effect of increased dairy protein consumption on insulin sensitivity, a potential explanation for the epidemiological data demonstrating an inverse relationship between diary consumption and CVD risk factors. Second, we will determine if this diet in subjects with metabolic syndrome results in an improvement of CVD risk factors. Finally, we will examine the effects of a higher carbohydrate and lower protein diet, indicative of individuals with metabolic syndrome and representative of a common American intake[35], on insulin sensitivity and CVD risk factors. The vigilance and thorough metabolic feeding inherent in this design will enable a clear distinction between dietary patterns and allow the determination of metabolic changes associated with each.

6.5 Study Procedures

6.5.1 Stable isotope studies for determination of glucose kinetics

Two peripheral catheters will be inserted in arm veins; one for infusion of a stable isotope of glucose, the other for blood sampling. Blood samples will be collected during the 4-hr stable isotope infusion study as shown in the figure below. Oral glucose test product is obtained from Fisher HealthCare (75 SUN-DEX®, Houston, TX). Stable isotopes will be obtained as sterile powders (Cambridge Isotope Labs, Andover, MA), and they will be compounded and dispensed by the UAMS Research Pharmacist in Little Rock; AR. Subjects will ingest a small amount of a different stable isotope of glucose with their SUN-DEX, in order to evaluate the contribution to plasma glucose from the exogenous glucose test solution.
Our primary endpoints will be glucose rate of appearance (Ra), glucose disposal (Rd), and insulin secretion. These data will allow us to determine the variable impact of glucose ingestion on the hepatic and peripheral mechanisms that may be responsible for potential improvements

in glycemia. Indexes of β-cell function will be computed as the ratios of area under the curve (AUC) $AUC_{insulin}$ to $AUC_{glucose}$ and the change in insulin over 30 min to the glucose at 30 min ($\Delta insulin_{30–0min}$ to $glucose_{30min}$)[36]. In addition, c-peptide will be measured to assess insulin secretion. In order to compare our studies with previous investigations, we will also determine the glucose AUC and the insulin responses to the OGTT. Infusion rate of the (6,6,$^2H_2$) glucose stable isotope is based upon subject's mass. Ingestion of the 1-^{13}C glucose isotope is concurrent with the oral glucose test drink as described by Basu et al.[37]. Volume of the ingested glucose stable isotope is based upon the subject's mass. See Figure 2 for an overview of the metabolic study.

Figure 2: Infusion Study Diagram

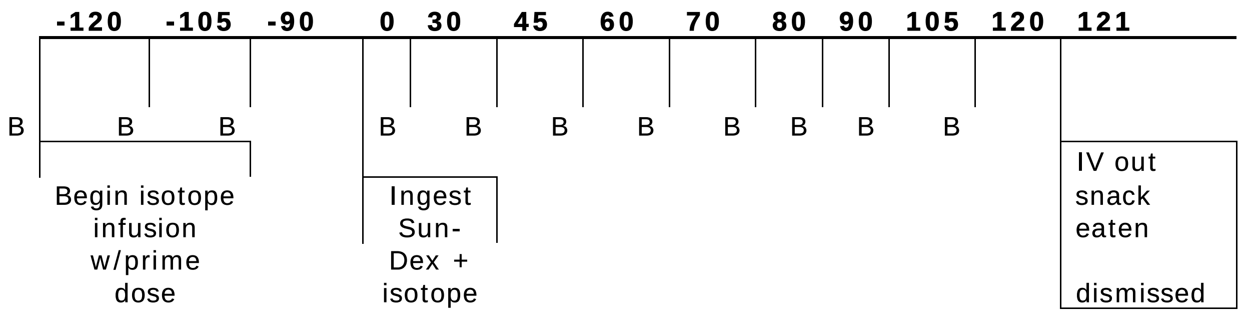

B= Blood sample

6.5.2 Whole body DEXA scan

Subjects will undergo two whole-body DEXA scans; one at the screening visit (1) and the second at visit 11. These scans will allow us to determine any changes in body composition as well as allow comparisons between subjects using % lean body mass as the units of expression. Scans will be done in the UAMS RIOA by study staff at no cost to subjects.

6.5.3 Blood sampling

Study staff will draw approximately 5 mL of venous blood during the screening visit to measure lipid panel and HbA1c. Approximately 88 mL will be drawn during each stable isotope/OGTT study. Total amount drawn over the estimated 5-week participation time frame is approximately 181 mL.

6.6 Subject Compensation

Subjects receive financial compensation in the form of up to a $325 check for participation in the study. Payment will be prorated as: $25 for visit 1, $50 for visit 2, $25 each for visits 3-10, and $50 for visit 11. Subjects will be mailed their check within 2-3 weeks after their participation ceases, regardless of whether they completed all visits.

6.7 Blinding

Most subjects will likely recognize the protein/carbohydrate content of the diet to which they have been assigned; therefore, blinding is not feasible. However, samples will be coded to prevent analytical bias.

6.8 Subject compliance

Compliance and caloric intake will be determined by supplying subjects with a point-and-shoot digital camera and instructions to photograph each meal before and after consumption. Subjects will also be given instructions to not rinse/clean food containers. Caloric intake will then be calculated and/or adjusted by the Research Dietician after each week's container return. In addition, container return and pre-post meal photos will help ensure adequate compliance. If subjects do not consume ≥ 80% of provided meals, they will be judged non-compliant and replaced with another subject. In the past, we have been successful in maintaining a high level of subject compliance (> 80%) by utilizing several techniques. First, we counsel each subject during the phone screening and outline each detail of the study protocol, including randomization to groups. Subjects must agree to all facets of the study protocol prior to enrollment. Second, each subject is provided with a personal calendar that outlines each specific detail of the study and their responsibilities as a volunteer. This calendar includes all procedures, food pick-up times and locations, and the corresponding dates and times for each procedure. This outline is developed according to the study protocol and in conjunction with the TRI dietary staff. Nonetheless, it is important to mention that subject preferences associated with time of day for food pick-up are discussed prior to enrollment and included in the outline. In addition, the office and cellular phone numbers of investigators are included on the consent form and the study outline. The above-mentioned factors and the regular interaction between investigators, staff and the subjects have resulted in ~80% compliance to dietary control in previous studies[34, 38].

6.9 Subject Recruitment

Subjects will be recruited using four separate methods. First, IRB-approved flyers will be posted in public areas around the Little Rock, AR area including the UAMS campus. Second, the Research Match online tool will be utilized in conjunction with the UAMS TRI staff. Third, we will call past subjects who have previously agreed to be contacted about future studies. Lastly, we will use the UAMS Data Warehouse to search for eligible subjects as defined below.
Step 1: After IRB permission is obtained to use the database, we will submit a query to the database to provide study staff with a list of patients who meet the following criteria:

- Ages 45-75 AND
- Systolic blood pressure >140
- Diastolic blood pressure >90
- Triglycerides > 130 mg/dl
- Glucose > 100 mg/dl

Step 2: With the list of results, study staff will contact (via UAMS email) the patient's PCP as documented in Centricity. If a PCP is not listed, staff will contact the physician who has been treating the patient for the majority of the most recent visits. Study staff will ask the physician if they would mail or otherwise provide their patient an introductory letter about this study (see Appendices).

Step 3: Patients that were provided the introductory letter who are interested in this study can then contact the study coordinator who will speak with them in detail about this study.

7.0 Analytical Methods

7.1 Blood plasma analyses

Glucose enrichment in blood will be performed by GCMS, and is well-established in our laboratory. Blood lipids will be measured on our automated clinical analyzer (Cobas C 111, Roche). Insulin, C-peptide (to determine insulin secretion), CRP, TNF-α, and IL-6 will be determined by ELISA.

7.2 Statistical Analysis

Demographic and metabolic characteristics will be summarized and tabulated by group using means, standard deviations, medians and ranges for continuous variables, while counts and percentages will be used for categorical variables. To address specific aim 1, Hotelling's T2 test will be used to compare the metabolic profile of subjects randomized to the Americanized diet to that of subjects in the high dairy diet. In this case, the metabolic profile will consist of the following three measures determined during the dual tracer oral glucose tolerance test: glucose disposal, glucose production and insulin secretion. We will also use the Hotelling's T2 to compare the groups with respect to the pre-diet and post-diet changes in metabolic profiles. If the Hotelling's test is found to be significant, Student's two-sample t-tests will be used to compare the groups with respect to each component. To address specific aim 2, we will also use Hotelling's T2 to compare blood lipid profiles and pro-inflammatory profiles of the groups. An α-level of 0.05 will be used to determine the statistical significance of the Hotelling's T2 tests. Sime's procedure will be used to adjust p-values for multiple comparisons when individual profile components are tested using Student's t-tests.

7.3 Power and Sample Size

The Hotellings T2 test will have approximately 85% power to detect effect sizes of 1.71 or larger in the Matsuda insulin sensitivity index (ISI; Figure 1B) when 12 evaluable subjects are enrolled in each group and an α-level of 0.05 is used to determine the statistical significance. It is important to note that combined utilization of isotope methodology with the OGTT to determine the rates of glucose appearance and disappearance is a more precise method of evaluating insulin sensitivity than the Matsuda index39. It will also allow us to distinguish changes in hepatic and peripheral glucose metabolism.

In terms of our secondary outcome measures, the Hotellings T2 test will have approximately 84% power to detect effect sizes of 47 mg/dl or larger in plasma triglycerides when 12 evaluable subjects are enrolled into each group and an alpha level of 0.05 is used to determine statistical significance. The effect size for the Hotelling's T2 is simply the difference between group profile means adjusted by the variance-covariance matrix of the profile components.

7.4 Interim Analyses

Analyses of dietary compliance will be an ongoing process by study staff. Analyses of blood samples will take place in batch format to minimize analytical error and variability. The PI will review data as it becomes available. Data will be documented on case report forms.

7.5 Data Storage

Source documents and CRFs will be stored in a secure area of the PI's laboratory. Access will be limited to study personnel. Documents will be archived according to UAMS policies regarding destruction of research records. At no time shall Protected Health Information be released to non-study personnel. Electronic data will be kept on an UAMS server that is password protected and is only accessible by study personnel.

7.6 Sample Storage

Blood samples will be kept frozen at -80 degrees Centigrade or colder once the initial processing has taken place. Samples shall be stored in appropriate freezers in the PI's laboratory, located in the UAMS IOA building. Said freezers are monitored continuously for proper temperature and working condition. Samples will be destroyed only after all data has been analyzed and reported to the sponsor. All blood samples shall be identified using a unique study acronym. None of a subject's personal identifiers shall be present on any biological sample.

8.0 Safety Reporting

8.1 Definitions

ICH-GCP definition: An *Adverse Event* is any untoward medical occurrence in a patient or clinical trial subject administered a study product and which does not necessarily have a causal relationship with this product.

A Serious Adverse Event (SAE) is any untoward medical occurrence or effect that, after intake of any amount of study product:

- Results in death.
- Is life-threatening (at the time of the event).
- Requires hospitalization or prolongation of existing inpatients' hospitalization.

- Results in persistent or significant disability or incapacity.
- Is a congenital anomaly or birth defect.

8.2 (Serious) Adverse Events handling

Any clinical study event that is judged to be an (S)AE, either reported spontaneously by the subject or observed by the investigator or his staff, is recorded on the (S)AE form during the course of the study. The investigator must ensure that this information, including onset, duration and nature of event, severity, and action taken, is captured.

The severity of any (S)AE is scored as follows:

- Mild: transient or mild discomfort; no medical intervention/therapy required.
- Moderate: mild to moderate limitation in activity; some assistance may be needed; and/or minimal medical intervention/therapy required.
- Severe: marked limitation in activity; some assistance usually required; and/or significant medical intervention/therapy/hospitalisation required.

The relationship of the (S)AE to the study product is assessed as being not related/unlikely/possibly/probably/ definitely related.

8.3 (S)AE reporting

Serious adverse events must be reported to the UAMS Institutional Review Board (IRB) 48 hours (2 working days) after their discovery. AEs will be reported in aggregate at routine intervals, e.g. Continuing Review, etc.

8.4 Follow-up of (S)AEs

All (S)AEs are followed by the investigator until they have abated, or until a stable situation has been reached. Depending on the event, follow up may require additional tests or medical procedures as indicated, and/or referral to the general physician or a medical specialist.

8.5 Risks/Benefits

There are no special benefits for the subjects apart from the possibility to get knowledge of their own results of the assessments at the screening visit. Expected risks associated with this

protocol are described in detail below. All experimental procedures will be performed by appropriately trained and credentialed personnel. IRB-approved SOPs will be used for the infusion procedures.

8.5.1 Peripheral catheter placement:

Briefly, the risks related to the catheter placement include pain, bleeding, bruising, risk of infection and vaso-vagal response. Some individuals have a sudden drop in blood pressure in response to the placement of catheters (vasovagal response). Symptoms may include light-headedness, nausea and possibly vomiting. There are no long-term effects associated with this response. In our experience, symptoms occur in 3.3% of our subjects. All subjects experiencing this response will be examined by a physician and given the option of discontinuing the study. If the physician and investigators find no reason to stop the study and the subject wishes to continue, the subject will be able to finish the study.

8.5.3 Blood sampling:

Blood samples will be collected solely for the purpose of experimentation. The blood will be used to measure the enrichment of the infused stable isotopes and the concentrations of glucose and insulin. About 181 mL of blood will be drawn during the entire study (including screening appt). This volume is much less than a blood donation and should not produce any noticeable effects.

8.5.4 Stable isotope infusion:

Briefly, the risk related to the isotope infusion is pyrogenic response.
All infusions are prepared by a licensed pharmacist at UAMS. The IRB-approved SOP for infusion procedures will be utilized. All stable isotopes are tested for sterility and pyrogenicity before the infusion study. The stable isotopes will be filtered during infusion through a sterile 0.22 micron (Millipore) filter placed in the infusion line. Stable isotopes are naturally occurring compounds and are not radioactive and are already present in the body in varying amounts. The infusion will be in a 'tracer' dose, i.e., a dose only detectable by GC/MS. Infusion of these molecules will increase the level of naturally

occurring isotopes by 7-10%. Any adverse reactions during the isotope infusion that suggest allergic reaction or infection (urticaria, flushing, nausea, vomiting, sweating, chills, altered heart rate, hypo/hypertension, and hyperthermia) will be promptly addressed by the study physician. Depending on the seriousness of the reaction, the infusion study will be terminated.

8.5.5 Stable Isotope ingestion:

Ingestion of approximately 4g of a stable isotope of glucose during each OGTT should pose no risk to subjects. This isotope will also be compounded and dispensed by the UAMS research pharmacist and is indistinguishable from normal glucose in the body.

8.6 Informed Consent Process

This study will be conducted in accordance with all applicable government regulations and University of Arkansas for Medical Sciences research policies and procedures. This protocol and any amendments will be submitted and approved by the UAMS Institutional Review Board (IRB) to conduct the study.

The formal consent of each subject, using the IRB-approved consent form, will be obtained before that subject is submitted to any study procedure. All subjects for this study will be provided a consent form describing this study and providing sufficient information in language suitable for subjects to make an informed decision about their participation in this study. The person obtaining consent will thoroughly explain each element of the document and outline the risks and benefits, alternate treatment(s), and requirements of the study. The consent process will take place in a quiet and private room, and subjects may take as much time as needed to make a decision about their participation. Participation privacy will be maintained and questions regarding participation will be answered. No coercion or undue influence will be used in the consent process. This consent form must be signed by the subject or legally acceptable surrogate, and the individual obtaining the consent. A copy of the signed consent will be given to the participant, and the informed consent process will be documented in each subject’s research record.

9.0 Use of Data

Study data and interpretation of results will be sent to DRI. Given the pervasive problem of sarcopenia and functional disability in older individuals, recommendations concerning varied protein intake pattern or increased protein intake represent an economical and practicable solution. Results may provide a strong scientific basis for recommending increased total dietary protein consumption. This evidence will support the recommendation of increased dairy consumption, because dairy ingredients are an excellent source of dietary protein that can conveniently be eaten at breakfast and lunch, as well as dinner.

Results will be disseminated to the sponsor (DRI) via a final technical report. The PI has the option to submit results for publication in scientific literature.

10.0 References

1. Wolfe RR, Miller SL, Miller KB. Optimal protein intake in the elderly. Clin Nutr 2008; 27(5):675-84. http://www.ncbi.nlm.nih.gov/entrez/query.fcgi?cmd=Retrieve&db=PubMed&dopt=Citation&list_uids=18819733
2. Lamarche B. Review of the effect of dairy products on non-lipid risk factors for cardiovascular disease. J Am Coll Nutr 2008; 27(6):741S-6S. http://www.ncbi.nlm.nih.gov/entrez/query.fcgi?cmd=Retrieve&db=PubMed&dopt=Citation&list_uids=19155434
3. Tholstrup T, Hoy CE, Andersen LN, Christensen RD, Sandstrom B. Does fat in milk, butter and cheese affect blood lipids and cholesterol differently? J Am Coll Nutr 2004; 23(2):169-76. http://www.ncbi.nlm.nih.gov/entrez/query.fcgi?cmd=Retrieve&db=PubMed&dopt=Citation&list_uids=15047684
4. Huth PJ, Park KM. Influence of dairy product and milk fat consumption on cardiovascular disease risk: a review of the evidence. Adv Nutr; 3(3):266-85. http://www.ncbi.nlm.nih.gov/entrez/query.fcgi?cmd=Retrieve&db=PubMed&dopt=Citation&list_uids=22585901
5. Khan NA, Hemmelgarn B, Herman RJ, Rabkin SW, McAlister FA, Bell CM, Touyz RM, Padwal R, Leiter LA, Mahon JL, Hill MD, Larochelle P, Feldman RD, Schiffrin EL, Campbell NR, Arnold MO, Moe G, Campbell TS, Milot A, Stone JA, Jones C, Ogilvie RI, Hamet P, Fodor G, Carruthers G, Burns KD, Ruzicka M, dechamplain J, Pylypchuk G, Petrella R, Boulanger JM, Trudeau L, Hegele RA, Woo V, McFarlane P, Vallee M, Howlett J, Katzmarzyk P, Tobe S, Lewanczuk RZ. The 2008 Canadian Hypertension Education Program recommendations for the management of hypertension: part 2 - therapy. Can J Cardiol 2008; 24(6):465-75. http://www.ncbi.nlm.nih.gov/entrez/query.fcgi?cmd=Retrieve&db=PubMed&dopt=Citation&list_uids=18548143
6. Wang L, Manson JE, Buring JE, Lee IM, Sesso HD. Dietary intake of dairy products, calcium, and vitamin D and the risk of hypertension in middle-aged and older women. Hypertension 2008; 51(4):1073-9. http://www.ncbi.nlm.nih.gov/entrez/query.fcgi?cmd=Retrieve&db=PubMed&dopt=Citation&list_uids=18259007
7. Appel LJ, Moore TJ, Obarzanek E, Vollmer WM, Svetkey LP, Sacks FM, Bray GA, Vogt TM, Cutler JA, Windhauser MM, Lin PH, Karanja N. A clinical trial of the effects of dietary patterns on blood pressure. DASH Collaborative Research Group. N Engl J Med 1997; 336(16):1117-24. http://www.ncbi.nlm.nih.gov/entrez/query.fcgi?cmd=Retrieve&db=PubMed&dopt=Citation&list_uids=9099655
8. Craddick SR, Elmer PJ, Obarzanek E, Vollmer WM, Svetkey LP, Swain MC. The DASH diet and blood pressure. Curr Atheroscler Rep 2003; 5(6):484-91. http://www.ncbi.nlm.nih.gov/entrez/query.fcgi?cmd=Retrieve&db=PubMed&dopt=Citation&list_uids=14525682
9. Zemel MB, Sun X. Dietary calcium and dairy products modulate oxidative and inflammatory stress in mice and humans. J Nutr 2008; 138(6):1047-52.

http://www.ncbi.nlm.nih.gov/entrez/query.fcgi?cmd=Retrieve&db=PubMed&dopt=Citation&list_uids=18492832

10. Despres JP, Cartier A, Cote M, Arsenault BJ. The concept of cardiometabolic risk: Bridging the fields of diabetology and cardiology. Ann Med 2008; 40(7):514-23. http://www.ncbi.nlm.nih.gov/entrez/query.fcgi?cmd=Retrieve&db=PubMed&dopt=Citation&list_uids=18608131
11. Pittas AG, Lau J, Hu FB, Dawson-Hughes B. The role of vitamin D and calcium in type 2 diabetes. A systematic review and meta-analysis. J Clin Endocrinol Metab 2007; 92(6):2017-29. http://www.ncbi.nlm.nih.gov/entrez/query.fcgi?cmd=Retrieve&db=PubMed&dopt=Citation&list_uids=17389701
12. Beydoun MA, Gary TL, Caballero BH, Lawrence RS, Cheskin LJ, Wang Y. Ethnic differences in dairy and related nutrient consumption among US adults and their association with obesity, central obesity, and the metabolic syndrome. Am J Clin Nutr 2008; 87(6):1914-25. http://www.ncbi.nlm.nih.gov/entrez/query.fcgi?cmd=Retrieve&db=PubMed&dopt=Citation&list_uids=18541585
13. Pereira MA, Jacobs DR, Jr., Van Horn L, Slattery ML, Kartashov AI, Ludwig DS. Dairy consumption, obesity, and the insulin resistance syndrome in young adults: the CARDIA Study. Jama 2002; 287(16):2081-9. http://www.ncbi.nlm.nih.gov/entrez/query.fcgi?cmd=Retrieve&db=PubMed&dopt=Citation&list_uids=11966382
14. Liu S, Song Y, Ford ES, Manson JE, Buring JE, Ridker PM. Dietary calcium, vitamin D, and the prevalence of metabolic syndrome in middle-aged and older U.S. women. Diabetes Care 2005; 28(12):2926-32. http://www.ncbi.nlm.nih.gov/entrez/query.fcgi?cmd=Retrieve&db=PubMed&dopt=Citation&list_uids=16306556
15. Rice BH, Cifelli CJ, Pikosky MA, Miller GD. Dairy components and risk factors for cardiometabolic syndrome: recent evidence and opportunities for future research. Adv Nutr; 2(5):396-407. http://www.ncbi.nlm.nih.gov/entrez/query.fcgi?cmd=Retrieve&db=PubMed&dopt=Citation&list_uids=22332081
16. Paddon-Jones D, Wolfe RR, Ferrando AA. Amino acid supplementation for reversing bed rest and steroid myopathies. J Nutr 2005; 135(7):1809S-1812S. http://www.ncbi.nlm.nih.gov/entrez/query.fcgi?cmd=Retrieve&db=PubMed&dopt=Citation&list_uids=15987870
17. Pal S, Ellis V, Dhaliwal S. Effects of whey protein isolate on body composition, lipids, insulin and glucose in overweight and obese individuals. Br J Nutr 2010; 104(5):716-23. http://www.ncbi.nlm.nih.gov/entrez/query.fcgi?cmd=Retrieve&db=PubMed&dopt=Citation&list_uids=20377924
18. Pal S, Ellis V. The chronic effects of whey proteins on blood pressure, vascular function, and inflammatory markers in overweight individuals. Obesity (Silver Spring) 2010; 18(7):1354-9. http://www.ncbi.nlm.nih.gov/entrez/query.fcgi?cmd=Retrieve&db=PubMed&dopt=Citation&list_uids=19893505

19. Akhavan T, Luhovyy BL, Brown PH, Cho CE, Anderson GH. Effect of premeal consumption of whey protein and its hydrolysate on food intake and postmeal glycemia and insulin responses in young adults. Am J Clin Nutr 2010; 91(4):966-75. http://www.ncbi.nlm.nih.gov/entrez/query.fcgi?cmd=Retrieve&db=PubMed&dopt=Citation&list_uids=20164320
20. Zemel MB. The role of dairy foods in weight management. J Am Coll Nutr 2005; 24(6 Suppl):537S-46S. http://www.ncbi.nlm.nih.gov/entrez/query.fcgi?cmd=Retrieve&db=PubMed&dopt=Citation&list_uids=16373953
21. Huth PJ, DiRienzo DB, Miller GD. Major scientific advances with dairy foods in nutrition and health. J Dairy Sci 2006; 89(4):1207-21. http://www.ncbi.nlm.nih.gov/entrez/query.fcgi?cmd=Retrieve&db=PubMed&dopt=Citation&list_uids=16537954
22. Dougkas A, Reynolds CK, Givens ID, Elwood PC, Minihane AM. Associations between dairy consumption and body weight: a review of the evidence and underlying mechanisms. Nutr Res Rev 2011:1-24. http://www.ncbi.nlm.nih.gov/entrez/query.fcgi?cmd=Retrieve&db=PubMed&dopt=Citation&list_uids=21320381
23. Boon N, Hul GB, Stegen JH, Sluijsmans WE, Valle C, Langin D, Viguerie N, Saris WH. An intervention study of the effects of calcium intake on faecal fat excretion, energy metabolism and adipose tissue mRNA expression of lipid-metabolism related proteins. Int J Obes (Lond) 2007; 31(11):1704-12. http://www.ncbi.nlm.nih.gov/entrez/query.fcgi?cmd=Retrieve&db=PubMed&dopt=Citation&list_uids=17579637
24. Boirie Y, Dangin M, Gachon P, Vasson MP, Maubois JL, Beaufrere B. Slow and fast dietary proteins differently modulate postprandial protein accretion. Proc Natl Acad Sci U S A 1997; 94(26):14930-5. http://www.ncbi.nlm.nih.gov/entrez/query.fcgi?cmd=Retrieve&db=PubMed&dopt=Citation&list_uids=9405716
25. Frid AH, Nilsson M, Holst JJ, Bjorck IM. Effect of whey on blood glucose and insulin responses to composite breakfast and lunch meals in type 2 diabetic subjects. Am J Clin Nutr 2005; 82(1):69-75. http://www.ncbi.nlm.nih.gov/entrez/query.fcgi?cmd=Retrieve&db=PubMed&dopt=Citation&list_uids=16002802
26. Poudyal H, Panchal SK, Ward LC, Waanders J, Brown L. Chronic high-carbohydrate, high-fat feeding in rats induces reversible metabolic, cardiovascular, and liver changes. Am J Physiol Endocrinol Metab 2012. http://www.ncbi.nlm.nih.gov/entrez/query.fcgi?cmd=Retrieve&db=PubMed&dopt=Citation&list_uids=22436699
27. Panchal SK, Ward L, Brown L. Ellagic acid attenuates high-carbohydrate, high-fat diet-induced metabolic syndrome in rats. Eur J Nutr 2012. http://www.ncbi.nlm.nih.gov/entrez/query.fcgi?cmd=Retrieve&db=PubMed&dopt=Citation&list_uids=22538930

28. Chun MR, Lee YJ, Kim KH, Kim YW, Park SY, Lee KM, Kim JY, Park YK. Differential effects of high-carbohydrate and high-fat diet composition on muscle insulin resistance in rats. J Korean Med Sci 2010; 25(7):1053-9. http://www.ncbi.nlm.nih.gov/entrez/query.fcgi?cmd=Retrieve&db=PubMed&dopt=Citation&list_uids=20592898
29. Even PC, Nadkarni NA, Chaumontet C, Azzout-Marniche D, Fromentin G, Tome D. Identification of behavioral and metabolic factors predicting adiposity sensitivity to both high fat and high carbohydrate diets in rats. Front Physiol; 2:96. http://www.ncbi.nlm.nih.gov/entrez/query.fcgi?cmd=Retrieve&db=PubMed&dopt=Citation&list_uids=22203804
30. Wolever TM, Gibbs AL, Chiasson JL, Connelly PW, Josse RG, Leiter LA, Maheux P, Rabasa-Lhoret R, Rodger NW, Ryan EA. Altering source or amount of dietary carbohydrate has acute and chronic effects on postprandial glucose and triglycerides in type 2 diabetes: Canadian trial of Carbohydrates in Diabetes (CCD). Nutr Metab Cardiovasc Dis 2012. http://www.ncbi.nlm.nih.gov/entrez/query.fcgi?cmd=Retrieve&db=PubMed&dopt=Citation&list_uids=22397878
31. Swinburn BA, Boyce VL, Bergman RN, Howard BV, Bogardus C. Deterioration in carbohydrate metabolism and lipoprotein changes induced by modern, high fat diet in Pima Indians and Caucasians. J Clin Endocrinol Metab 1991; 73(1):156-65. http://www.ncbi.nlm.nih.gov/entrez/query.fcgi?cmd=Retrieve&db=PubMed&dopt=Citation&list_uids=2045466
32. Wilson GJ, Layman DK, Moulton CJ, Norton LE, Anthony TG, Proud CG, Rupassara SI, Garlick PJ. Leucine or carbohydrate supplementation reduces AMPK and eEF2 phosphorylation and extends postprandial muscle protein synthesis in rats. Am J Physiol Endocrinol Metab 2011; 301(6):E1236-42. http://www.ncbi.nlm.nih.gov/entrez/query.fcgi?cmd=Retrieve&db=PubMed&dopt=Citation&list_uids=21917636
33. Rieu I, Balage M, Sornet C, Giraudet C, Pujos E, Grizard J, Mosoni L, Dardevet D. Leucine supplementation improves muscle protein synthesis in elderly men independently of hyperaminoacidaemia. J Physiol 2006; 575(Pt 1):305-15. http://www.ncbi.nlm.nih.gov/entrez/query.fcgi?cmd=Retrieve&db=PubMed&dopt=Citation&list_uids=16777941
34. Coker RH, Williams RH, Yeo SE, Kortebein PM, Bodenner DL, Kern PA, Evans WJ. The impact of exercise training compared to caloric restriction on hepatic and peripheral insulin resistance in obesity. J Clin Endocrinol Metab 2009; 94(11):4258-66. http://www.ncbi.nlm.nih.gov/entrez/query.fcgi?cmd=Retrieve&db=PubMed&dopt=Citation&list_uids=19808853
35. Fulgoni VL, 3rd. Current protein intake in America: analysis of the National Health and Nutrition Examination Survey, 2003-2004. Am J Clin Nutr 2008; 87(5):1554S-1557S. http://www.ncbi.nlm.nih.gov/entrez/query.fcgi?cmd=Retrieve&db=PubMed&dopt=Citation&list_uids=18469286
36. Stumvoll M, Mitrakou A, Pimenta W, Jenssen T, Yki-Jarvinen H, Van Haeften T, Renn W, Gerich J. Use of the oral glucose tolerance test to assess insulin release and insulin sensitivity.

Diabetes Care 2000; 23(3):295-301. http://www.ncbi.nlm.nih.gov/entrez/query.fcgi?cmd=Retrieve&db=PubMed&dopt=Citation&list_uids=10868854

37. **Basu R, Di Camillo B, Toffolo G, Basu A, Shah P, Vella A, Rizza R, Cobelli C. Use of a novel triple-tracer approach to assess postprandial glucose metabolism. Am J Physiol Endocrinol Metab 2003; 284(1):E55-69. http://www.ncbi.nlm.nih.gov/entrez/query.fcgi?cmd=Retrieve&db=PubMed&dopt=Citation&list_uids=12485809**
38. **Coker RH, Hays NP, Williams RH, Brown AD, Freeling SA, Kortebein PM, Sullivan DH, Starling RD, Evans WJ. Exercise-induced changes in insulin action and glycogen metabolism in elderly adults. Med Sci Sports Exerc 2006; 38(3):433-8. http://www.ncbi.nlm.nih.gov/entrez/query.fcgi?cmd=Retrieve&db=PubMed&dopt=Citation&list_uids=16540829**
39. **Hays NP, Starling RD, Sullivan DH, Fluckey JD, Coker RH, Evans WJ. Comparison of insulin sensitivity assessment indices with euglycemic-hyperinsulinemic clamp data after a dietary and exercise intervention in older adults. Metabolism 2006; 55(4):525-32. http://www.ncbi.nlm.nih.gov/entrez/query.fcgi?cmd=Retrieve&db=PubMed&dopt=Citation&list_uids=16546484**

11.0 Appendices

11.1 Medical History and Physical Examination

Study #:__________ **Subject ID #:** ___________ **Initials:** _____ **Date:** _______________

General Health: __________________________
PMHx:____

T HR RR BP / Ht__________ Wt ________ BMI ________		
**Meds:**		
**Allergies:**		

Smoking:____ PPD x____Yrs. EtOH:________

**ROS:** fever chills sweats wtΔ fatigue appetite sleep	GI: dysphagia N / V / D / C abd pain GERD hematochezia jaundice
Skin: itching rash sores susp. moles/lesions- healing recentΔ	GU: freq urgency hesitancy dys- hematuria incont UTI's stones
Head: dizzy fainting HA/LOC trauma	Genital: testicular masses hernias
Eyes: correction Δvision-double tearing itching/redness	Endocrine: polyuria polydipsia skin/hair Δ thyroid hx
Ears: Δhearing ringing earache vertigo/tinnitus	Vascular: claudication DVT hx
Nose: epistaxis rhinorrhea allergies	MSK: jt pain stiffness arthritis gout
Mouth/Throat: bleeding gums sore mouth/throat swollen neck	Neuro: numbness weakness/atrophy seizure/tremor
CV: angina palpitations DOE orthopnea/PND edema	Psych: depression anxiety recent memoryΔ
Pulm: SOB wheeze cough hemoptysis TB	Female: regular dysmenorrhea pregnancies menopause
Hematologic: bruise /bleed easily transfusion hx	Breast: skinΔ lumps pain discharge

**Physical Exam:** Gen: Well	Skin: cap refill: ____ no rash lesions ___________________
Head: no trauma no bruising no masses<br>Eyes: PERRLA EOMI no ptosis sclera clear<br>Ears: good acuity TM: nl reflex/intact<br>Nose: nl<br>Mouth/Throat: nl/pink, moist mucous membranes no lesions	Neurological: Alert & oriented×3, nl MS via conversation<br>Cranial Nerves: II - XII intact/nl<br>Motor: 5/5 UE/LE's bil<br>Sensation: intact LT UE/LE's<br>DTRs: symmetric/nl biceps knee ankle
Neck: no LAD no masses no bruits no JVD supple stiff	Abdomen: soft, NT/ND BS + no masses / organomegaly
Chest: CTA bil equal expansion	Extremities: no C/C/E Major jts: no swelling full ROM
Heart: Reg no M/R/G	Pulses Bil: PT / DP: 2+

Notes
____
____

Eligible: Yes / No Physician Signature: ___________________________

11.2 Stable Isotope Infusion SOP

Policy: The following information is written to serve as a resource and guideline for the stable isotope infusion procedure. This SOP will ensure that the procedure is performed in a consistent manner that protects participant safety.

Purpose: The stable isotope infusion procedure is conducted to measure in-vivo substrate kinetics (amino acid, glucose, fatty acids, etc) (1).

Responsibility and Accountability:
Principal Investigator: Will oversee, direct, and be responsible for assuring adherence to the entire procedure.
Study Physician: Responsible for overall medical supervision of the procedure. Will sign order(s) for related procedures/supplies, including infusates.
On-call physician: Will be on call throughout the procedure and available to respond to any emergencies. Depending upon the facility used for the procedure, the on-call physician(s) will be different individuals.
Study/staff nurse: Will provide immediate supervision throughout the procedure. Responsible for administering correct compound in correct dose to correct subject, as ordered (2).
Pharmacist: Will prepare infusates *per* physician's order.

Definitions:

Stable isotope – a non-radioactive variant (heavier by electron mass) of a naturally-occurring substance, e.g. glucose, amino acids, etc.

Materials:

1. Equipment: Calibration records for infusion pumps used will be maintained according to institutional policy.
2. All infusates will be prepared by a licensed pharmacist. Any drug to be infused (i.e. insulin or any other hormone) will have a written prescription by the study physician.

Pre-Procedure:

Personnel will document and record time for the following on the procedure flowsheet:

1. Verify volunteer has already completed the consent and screening process.
2. Nurse documents subject's vital signs, verifies that subject has fasted as required by protocol, and has met any other protocol-specific requirements and notes this in the record.
3. Nurse inserts blood sampling catheter and draws baseline sample and records these events in the record.
4. Nurse inserts infusion line catheter.
5. All syringes containing infusates will be labeled by the institutional pharmacy for subject name, medication/isotope, volume, date and initials.
6. A licensed medical person must confirm and document the placement and setup of infusates on the study flowsheet.
7. The study physician will be notified of the date and time for each procedure and will usually be available by pager or cell phone throughout the procedure.
8. If the study physician is not available or for a medical emergency, the on-call physician will be available by pager or cell phone.
9. A nurse will be responsible for maintenance of all catheters and be present during the procedure.

Procedure:

1. The subject will be advised to rest in a recumbent and/or supine position for the procedure.
2. Blood and muscle samples will be obtained according to the study-specific flowsheet (attached per submission). Blood samples will be obtained by licensed medical personnel or specifically trained and credentialed technical staff. Muscle biopsies will be obtained according to the SOP "Good Clinical Practices for Muscle Biopsies on Research Participants" by UAMS physicians.
3. Subjects will be monitored for adverse reactions to the infusion and blood and muscle sampling by study personnel and nurse. Any adverse reactions will be reported to the study physician or on-call physician immediately. If a reaction is suspected to be attributed to the infusion, it will be stopped and a sample preserved for later quality testing.
4. Subjects will be asked to comment on their general well-being during the procedure.

Post Procedure and Discharge:

1. Immediately following the completion of the procedure, each subject (for fasted studies) will receive a meal and will be encouraged to consume the meal in its entirety.

Emergency Procedures and Adverse Event Reporting:

All stable isotope infusion procedures will be performed as described in this SOP to minimize the risk of an unanticipated event. These procedures will be performed in the CRC at UAMS Central hospital.

Emergencies will be handled according to institutional guidelines. Adverse events will be reported by the PI to all relevant regulatory bodies (IRB, sponsor) pertaining to the specific protocol. Incident reports will be filed according to individual institutional guidelines.

Documentation: The study nurse will sign the study flowsheet, which will be reviewed by the PI at the completion of the study. Documentation for outpatients will be according to institutional policy.

Sources/References: All drug products for any study shall be prepared in accordance with Arkansas Law (Regulation 07-02-0001; Drug Products and Descriptions; Standards for Compounding and Dispensing Sterile Products).

1. Wolfe RR and Chinkes DL. Isotope Tracers in Metabolic Research: Principles and Practice of Kinetic Analysis. Wiley, New York, New York, 2004, 274 pages.

2. Deglin JH and Vallerand AH. Davis's Drug Guide for Nurses. 9th ed. F.A.Davis, Philadelphia, PA, 2005, xx-xxii.

11.3 Advertisement

UAMS

DONALD W. REYNOLDS
INSTITUTE ON AGING

UNIVERSITY OF ARKANSAS FOR MEDICAL SCIENCES

**HEALTHY VOLUNTEERS NEEDED FOR
A NUTRITIONAL STUDY**

The Center for Translational Research in Aging and Longevity at UAMS is recruiting overweight volunteers 45-75 years of age for a study to measure if blood sugar control improves by eating a specific diet for four weeks.

The study will involve a one-hour screening visit, 8 brief visits to retrieve your meals, and two 5-hour visits for testing. We will collect blood samples at 3 visits. Participants will get up to $325 for their participation. If interested, please call 501-526-5701 for more information.

This study is supported by the Dairy Research Institute.

526-5701 DAIRYMAC study
526-5701 DAIRYMAC study
526-5701 DAIRYMAC study
526-5701 DAIRYMAC study
526-5701 DAIRYMAC study
526-5701 DAIRYMAC study
526-5701 DAIRYMAC study
526-5701 DAIRYMAC study
526-5701 DAIRYMAC study
526-5701 DAIRYMAC study
526-5701 DAIRYMAC study
526-5701 DAIRYMAC study
526-5701 DAIRYMAC study
526-5701 DAIRYMAC study
526-5701 DAIRYMAC study
526-5701 DAIRYMAC study
526-5701 DAIRYMAC study

11.4 Introductory Letter to Potential Subjects

UAMS
DONALD W. REYNOLDS INSTITUTE ON AGING
UNIVERSITY OF ARKANSAS FOR MEDICAL SCIENCES

<Date>

<Name>
<Address1>
<Address2>

Dear <Name>,

I am writing to let you know about a research study which is being conducted by Arny Ferrando, Ph.D., Robert Coker, Ph.D., and Gohar, Azhar, M.D., at the University of Arkansas for Medical Sciences. I am contacting you because you might be eligible for this study.

This study is being conducted to determine whether a specific diet will improve how your body controls blood sugar levels.

We are looking for participants who want to take part in this research study. Specifically, we are looking for people who are:

- Men and women aged 45-75;
- BMI > 30

If you decide to take part in this research, we would:

- Review the study with you, at the Reynolds Institute of Aging, University of Arkansas for Medical Sciences.
- At your convenience, your involvement in this study will involve the following:
    - Screening Visit
        - Go over Consent and HIPAA
        - DEXA Scan (low-radiation X-ray of bone, muscle, and fat mass)
        - Physical examination (done by a study physician)
        - Fasting blood draw
    - Two metabolic studies (4.5 hours long; one prior to and one at the end of the 28 days of meals)
    - Eight visits to collect meals over the course of 4-weeks (Tuesdays and Fridays)

There is no cost to participate in the study.

As a thank you for taking part in the study, you would receive up to $325 at the conclusion of your participation in the study.

Within one to two weeks of receiving this letter, our Study Coordinator Cosby Lasley will call you. At that time please let her know if you are interested in hearing more about the study.

Letter for Prospective Participants
Dairy Macronutrient Effects on the Metabolic Syndrome
Date: 13 December 2013
Version 2.0

UAMS
DONALD W. REYNOLDS INSTITUTE ON AGING
UNIVERSITY OF ARKANSAS FOR MEDICAL SCIENCES

Taking part in research is voluntary. You and your family may choose not to participate. If you decide not to take part in this study, your decision will have no effect on any care you receive at UAMS.

We strive to provide the best possible patient care and we maintain the same principles in the conduct of research. If you participate in the study and have any concerns or discomfort at all, please don't hesitate to contact your primary care physician. You can do so by calling the clinic at 501-686-6219. You may also withdraw from the study at any time, and you will not be required to give a reason for withdrawing.

Sincerely,

Arny Ferrando, Ph.D.
Principal Investigator
Professor, UAMS Geriatrics
501-526-5711

Letter for Prospective Participants
Dairy Macronutrient Effects on the Metabolic Syndrome
Date: 13 December 2013
Version 2.0

11.5 Diet Diary

UAMS Food Record

Subject ID#: ________ ________________

Day & Date of Record: ______________________________

Time	Meal*	Place*	Food/Beverage Item & Description	Preparation Method	Amount	Office Use Only

*Meal Codes:
B Breakfast
L Lunch
D Dinner
S Snack

*Place Codes:
H Home
R Restaurant (Please specify name of restaurant)
F Friends
W Work
